# Systematic review on prevalence of ADHD, possible ADHD or ADHD symptoms in medical students

**DOI:** 10.3389/fpsyt.2025.1684727

**Published:** 2025-12-02

**Authors:** Nicholas Yew Wen Lee, Melvyn Wei Bin Zhang

**Affiliations:** 1 MOH Holdings, Singapore, Singapore; 2 National Addictions Management Service, Institute of Mental Health, Singapore, Singapore

**Keywords:** attention deficit-hyperactivity disorder (ADHD), prevalence, disability, medical students, medical education

## Abstract

**Introduction:**

Attention-Deficit/Hyperactivity Disorder (ADHD) is a persistent neurodevelopmental disorder that often continues into adulthood, with a global adult prevalence of approximately 3.1%. Among medical students, the condition may be particularly underdiagnosed and undertreated, despite the intense cognitive, emotional, and psychological demands of medical education. This systematic review aimed to examine the prevalence of ADHD in medical students across diverse settings.

**Methods:**

A comprehensive literature search was conducted in September 2024 across seven major databases, yielding 499 articles.

**Results:**

After de-duplication and applying inclusion criteria, 29 studies from 17 countries were included, encompassing 24,578 medical students. Reported prevalence rates ranged widely from 1.7% (self-report) to 38.9% (ASRS screener), with substantial variation across countries and even within the same regions. The most commonly used diagnostic instrument was the World Health Organization Adult ADHD Self-Report Scale (ASRS), though studies used different cutoffs and subscales. Other methods included the Wender Utah Rating Scale, structured clinical interviews, and self-report measures. Studies relying on self-report tended to show lower prevalence rates compared to those using structured tools.

**Discussion:**

Higher prevalence rates suggest that the stressors of medical education may amplify ADHD symptoms or that these environments attract or select for individuals with such traits. Despite the significant prevalence and associated functional impairments, there remains a lack of structured support systems for affected students. Early identification of ADHD in medical training could allow for tailored interventions, improved academic performance, reduced burnout, and better long-term outcomes. Additionally, the under-recognition of ADHD in medical students may have downstream effects on patient care if symptoms persist into clinical practice. This review underscores the need for standardized diagnostic criteria, structured assessments, and greater institutional support to address the challenges posed by ADHD in medical education. Further research should explore longitudinal outcomes, treatment efficacy, and the development of comprehensive support strategies for medical students with ADHD.

## Introduction

Attention-Deficit/Hyperactivity Disorder (ADHD) is a neurodevelopmental condition characterized by persistent patterns of inattention, hyperactivity, and impulsivity that interfere with functioning or development (1). While often associated with childhood, adult ADHD has an estimated worldwide prevalence of 3.1% based on a previous umbrella review (2). It is significantly underdiagnosed, and undertreated leading to long term health, personal, social, and economic costs and suffering (3, 4). Compounding the difficulties are the challenges in its evaluation in adulthood (5, 6).

The most widespread screening tools for the diagnosis of ADHD is the World Health Organization (WHO) Adult ADHD Self-Report Scale (ASRS). It consists of a 6-question screener (4 or more symptom count yielding a positive screening) and an extended scale with the 18 symptoms of ADHD on a 5-point Likert scale with a score of 0-72. The 6-question screener had a sensitivity of 68.7%, specificity of 99.5% and total classification accuracy of 97.9% when compared against DSM-IV. It was shown that the 6 question screener outperformed the unweighted 18 question ASRS (7). Other screening tools include the Wender Utah Rating Scale (WURS) and Connor’s Adult ADHD Rating Scale (8). There are also structured diagnostic interviews including the Diagnostic Interview for ADHD in Adults (DIVA) or the Connors Adult ADHD Diagnostic Interview (9, 10).

Medical training is cognitively and emotionally demanding, and medical students are known to report higher levels of psychological distress, and mental health conditions than their same-aged peers (11–13). Studies have reported that ADHD in medical students is common and causes disability in medical students (14–18). It has been associated with academic underperformance, increased stress, anxiety, and depression levels, and higher levels of burnout (19–22). As physicians, ADHD resulted in challenges with work performance through increased medical errors, challenges with progression in residency and examinations (23). On top of these significant occupational impairments, there were impairments in personal and interpersonal realms (20, 24). In a study of UK doctors seeking mental health help, over 1/3 (35%) screened positive for ADHD using the ASRS questionnaire, and these doctors had high degrees of comorbid mood and anxiety disorders (25). Certain other professions report high prevalence of ADHD and its symptoms, such as lawyers (12.5%) (26), pharmacists (14.4%) (27), physician assistants (9.3%) (28) and nurses (4% self-reported, 20% who suspected they had the disease) (29).

Despite the challenges medical students and physicians with ADHD face, there is little professional support available (20). The underdiagnosis and undertreatment of ADHD in future physicians is concerning, and reducing this could have a real-world impact especially if life-threatening medical errors could be averted (4, 23, 30). There is a lack of studies on rates of medical errors in treated or untreated ADHD physicians. However, it has been shown that stimulant treatment of ADHD is associated with a reduction in serious transport accidents by 41-49% (31). Also, screening for ADHD may also allow medical schools to identify comorbid mental health conditions presenting with difficulties in attention (25).

There are currently no systematic reviews or meta-analysis of prevalence in this critical population to our knowledge. Several studies have explored the prevalence of ADHD in medical students, with findings varying significantly based on population, diagnostic criteria, and assessment methods. For example, research conducted in Italy reported self-reported ADHD prevalence rates as low as 0.6% (32), while studies in Thailand found rates as high as 30% (33). These discrepancies highlight the need for a systematic review to synthesize available evidence and identify patterns in prevalence to guide management in this population which is important in health provision.

This review aims to consolidate current knowledge on ADHD prevalence among medical students, identify gaps in the literature, and inform strategies for addressing ADHD in this population.

## Methods

This systematic review followed the Preferred Reporting Items for Systematic Reviews and Meta-Analyses (PRISMA) guidelines (34).

### Search strategy

An information specialist from the Lee Kong Chian School of Medicine, Nanyang Technological University, Singapore was consulted to assist in the refinement of the search terminologies and strategies. This was performed to ensure the necessary breadth of the search and that all the relevant articles were captured. We conducted a comprehensive search across databases including PubMed, Medline (Ovid), PsycInfo, ScienceDirect, Scopus, Web of Science, Cochrane Library using the following search strategy from database inception to September 2024:

(“attention deficit disorder with hyperactivity”[MeSH: NoExp] OR ADHD[Title/Abstract] OR ADDH[Title/Abstract] OR “Attention Deficit Disorder*”[Title/Abstract] OR “Attention Deficit Hyperactivity Disorder*”[Title/Abstract] OR “Attention Deficit-Hyperactivity Disorder*”[Title/Abstract] OR “Hyperkinetic Syndrome”[Title/Abstract] OR “Minimal Brain Dysfunction”[Title/Abstract]) AND (“students, medical”[MeSH: NoExp] OR “medical student”[Title/Abstract:~3] OR “medical students”[Title/Abstract:~3]). Grey literature was not included due to the anticipated low yield for prevalence studies in medical students.

Using this approach, a total of 499 articles were retrieved from the various database, of which 241 were duplicates.

### Inclusion and exclusion criteria

Only articles which were written in English were included. There was no restriction on study types allowed. Articles were included if there was a prevalence included of ADHD in medical students. Articles were excluded if the prevalence of ADHD was not available, or if the participants were not from an allopathic medical school (MBBS/MD-equivalent programs globally). These included populations such as college students, other faculties or osteopathic medical students.

### Selection of articles

Two authors (NL and MZ) independently selected the relevant articles. Articles were initially screened based on their titles and abstracts. Articles that were shortlisted were then further evaluated against the inclusion and exclusion criteria. If the reviewers disagreed, this was resolved through a discussion with the final selection being a consensus. The selection of the articles for inclusion was in accordance with the Preferred Reporting Items for Systematic Reviews and Meta-Analysis Guidelines.

### Statistical analysis

The following data were systematically extracted from each of the identified articles and recorded on a standardized electronic data collation form: (a) authors and study year, (b) study design and methodology (sample size, demographics of sample, (c) method of assessment of ADHD, (d) prevalence of ADHD.

As the reported outcome measures and methods of assessment were heterogeneous, a meta-analytical synthesis was not undertaken. Hence, a qualitative synthesis was undertaken instead. There was no risk of bias assessment as the intent of the study was to present the data qualitatively in a narrative manner.

### PRISMA flow chart

A flow chart illustrates the screening process, including the number of records identified, screened, and excluded, along with reasons for exclusion.

## Results

### Study characteristics

**Figure 1:**
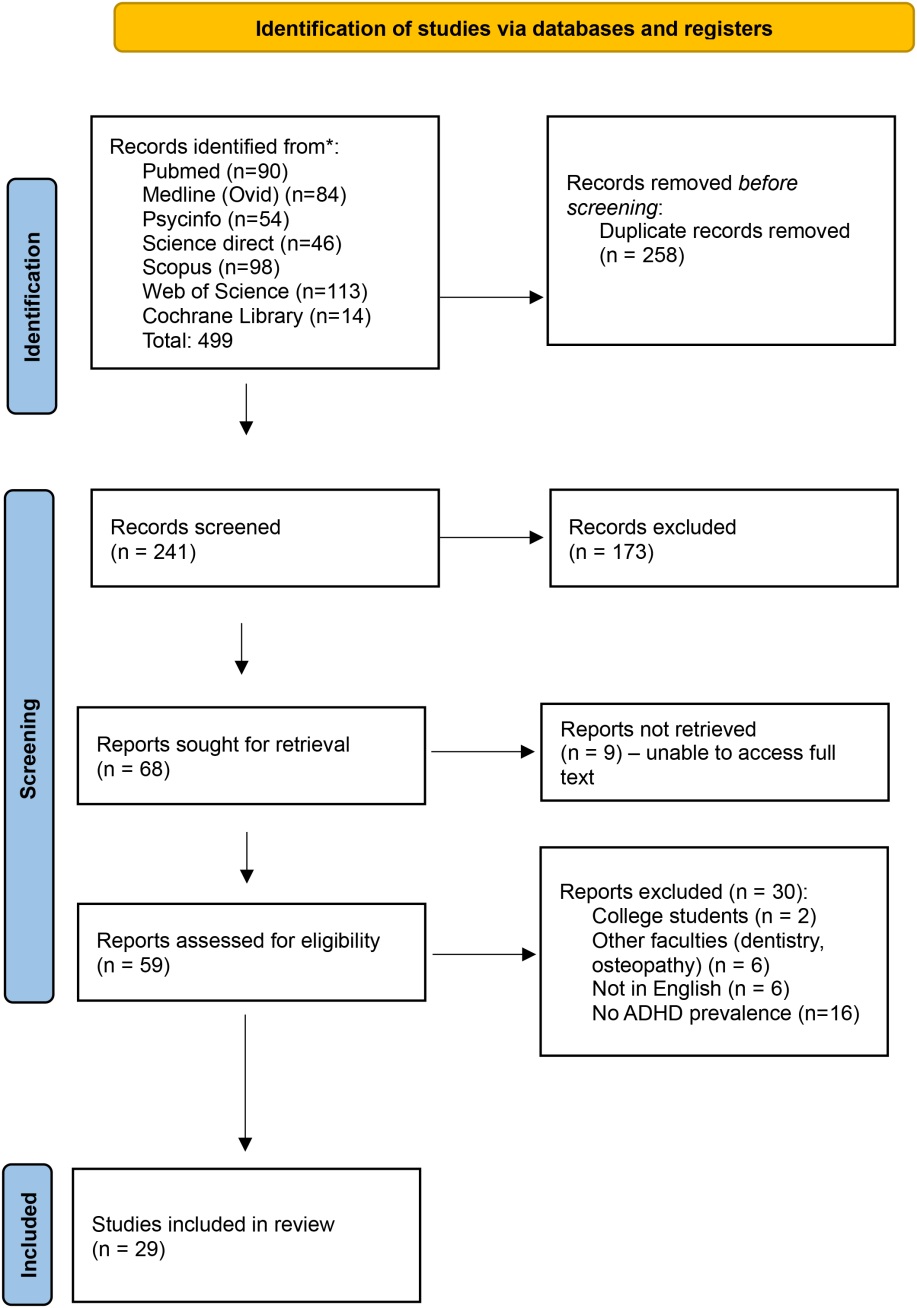
PRISMA flowchart.

Based on our search strategy, a total of 499 papers were identified from the 7 major databases. Following de-duplication, 241 abstracts were screened. 68 full text reports were sought, out of which 9 full-text articles were not available. Following review of full text, 29 studies were included in the final review with reasons for rejection as detailed in the PRISMA chart in **Figure 1**. The studies encompassing a total sample size of 24578 medical students from various countries, including Thailand, South Africa, Pakistan, the USA, and Iran. Sample sizes ranged from 98 to 5693 participants per study. **Table 1** provides an overview of the core characteristics of the included studies (n=29).

**Table 1:** List of studies and reported findings.

Name of study (year)	Sample size	Years of study	Percentage of males in population (%)	Mean age	Country	Method of assessment	Prevalence of ADHD (%)
Galván-Molina et al. (2017) (35)	323	1,3,6	53	21.45	Mexico	ASRS > 4 symptoms	27.9
Rapinesi et al. (2018) (36)	98	all	34.9	23.1	Italy	ASRS > 4 symptoms	18.4
						SCID	16.3
Mattos et al. (2018) (37)	662	all	47	23.6	Brazil	ASRS > 4 symptoms	37
						KSADS	7.9
						KSADS with probing	4.5
Cohen et al. (2015) (38)	229	all	43	26.42 ADHD patients (25.85)	Israel	ASRS > 4 symptoms	22.6
						Self-report	9.6
Kandre et al. (2020) (39)	427	all	54.6	No age data	India	ASRS > 4 symptoms	19.7
Watanabe et al. (2021) (40)	151	5	No gender data	23.7	Japan	ASRS > 4 symptoms	20.5
Mohamed et al. (2021) (41)	300	5	44	22.5	Egypt	ASRS > 4 symptoms	12.7
Batool et al. (2022) (42)	271	1-2	No gender data	No age data	Pakistan	ASRS > 4 symptoms	12.2
Karawekpanyawong et al. (2023) (43)	124	1	37.1	18.78 (ADHD patients 18.55)	Thailand	ASRS > 4 symptoms	25
Yingchankul et al. (2023) (33)	441	all	40.4	20.6	Thailand	ASRS > 4 symptoms	29.9
Shebl et al. (2023) (44)	990	All	No gender data	No age data	Egypt	ASRS > 4 symptoms	11
AlOmar et al. (2023) (45)	3219	all	42.2	22.18	Saudi Arabia	ASRS > 4 symptoms	38.9
Hajduk et al. (2024) (46)	503	all	No gender data	No age data	Germany	ASRS > 4 symptoms	13.3
Heidari et al. (2023) (47)	168	all	63.7	22.38	Iran	ASRS (criteria not specified)	20.2
Alsafar et al. (2024) (17)	354	2-7	29.7	21.8	Saudi Arabia	ASRS (criteria not specified)	26
Ahmed et al. (2024) (48)	979^1^	all	32.2	20.2	Egypt	ASRS > 17	28.2
Shen et al. (2018) (49)	5693	all	10.8	18.4	China	ASRS > 17 and WURS > 30	3.5
Shi et al. (2018) (50)	521	all	34.6	20.42	China	ASRS > 17 and WURS >46	8.4
Zafar et al. (2024) (51)	200	all	69	21.08	Saudi Arabia	ASRS > 17 and WURS >46	33.5
Ashor et al. (2012) (52)	361	all	58.7	21.4	Iraq	ASRS subscale > 13	16.6
Shen et al. (2020) (53)	4882	all	11	No age data	China	ASRS subscale (inattention) > 17	24.1
						ASRS subscale (hyperactivity) > 17	7.5
Tuttle et al. (2010) (54)	388	all	56	No age data	USA	Self-report	5.5
Bidwal et al. (2015) (28)	173^1^	all	59	27.2	USA	Self-report	4
Retief et al. (2016) (55)	251	2,4	73	20.9	South Africa	Self-report	2
Jain et al. (2017) (56)	541	all	52.7	No age data	South Africa	Self-report	2.96
Alrakaf et al. (2020) (57)	1177	all	51.1	No age data	Saudi Arabia	Self-report	3.3
Louw et al. (2022) (58)	253	all	46.6	No age data	South Africa	Self-report	2.4
Fasanella et al. (2022) (59)	263	all	33.5	22.9	Brazil	Self-report	3.8
Mansour et al. (2023) (60)	636	all	43.7	No age data	Jordan	Self-report	1.7

^1^Sample size of medical students only.

### Demographic data

The included participants ranged from all years of medical school, spanning 17 countries globally. 30.2% of the respondents were male (7193 of 23804) patients with available gender data) and mean age of the participants ranged from 18.4 to 27.2 years old.

### Diagnostic assessment methods

The diagnostic methods used are reported in **Table 2** below. The most common diagnostic tool was the ASRS screener (n=13). The next most common was self-report (n=10) This was followed by the full ASRS subscale, with 3 studies using it alone with different cutoffs (> 14 vs > 18) and 2 combining it with the WURS.

**Table 2:** ADHD assessment methods utilized.

Diagnostic assessment method and cut-off values	Number of studies reporting such data
ASRS screener (>4 symptoms)	13
ASRS subscale > 14 alone	1
ASRS subscale > 17 alone	1
ASRS subscale and WURS > 30	2
ASRS subscale > 17 and WURS > 46	1
ASRS screener (cutoff unclear)	2
Self-report	9
SCID	1
KSADS	1

### ADHD diagnosis rates

ADHD diagnostic rates varied widely across different methods of assessment, as well as countries. They ranged from the lowest rate of 1.7% (self-report) to 38.9% (ASRS screener). There was significant variance between studies done in the same country as well, likely contributed by different methods of assessment. The full list of studies and prevalence are listed below in **Table 1**.

## Discussion

### Rates of ADHD prevalence

This review is, to our knowledge, the first to systematically review prevalence estimates of ADHD specifically in medical students. This review encompasses multiple countries, diagnostic tools, cultures, and educational systems. While ADHD has been discussed within the broader literature on medical students with disabilities, a focused synthesis on ADHD prevalence has been lacking. Our sample was a representative global sample. Our findings show considerable variability in prevalence rates—from 1.7% (self-report) to 38.9% (ASRS screener)—reflecting not only differences in assessment methods, but also the impact of cultural attitudes, institutional policies, and healthcare system requirements. In some countries, a formal psychiatric diagnosis is required for students to access accommodations; in such settings, self-reported prevalence may underestimate true burden. Conversely, screening tools without clinical confirmation may overestimate prevalence by capturing difficulties with attention which may be transient or related to other mental health difficulties. Our review includes multiple studies which report ADHD prevalence rates higher than those observed in the general population, suggesting that the unique pressures of medical training might exacerbate symptoms. This is in keeping with other studies reporting higher levels of mental health distress and diagnoses in medical students. Quite strikingly, self-reported ADHD yielded much lower rates compared to validated screening and other diagnostic methods, and could suggest a lack of recognition should this be the main means through which medical students are identified for support.

### Subgroup populations

One critique may be that the sample was largely female, however, this is in keeping with the rising population of females in medicine and medical school (61–63). In adult ADHD, the estimated prevalence between genders is more comparable than in childhood ADHD, and hence the increased proportion of females is unlikely to skew the results (64). Studies with majority male populations did show similar prevalence figures. In our review, no formal subgroup analysis was conducted as no metanalysis was performed. There isn’t a clear trend that studies of majority male or female patients reported higher prevalence, the different assessment methods tend to account for the larger changes in prevalence. A minority of studies reported specific year data, which did not show any significant trends in prevalence rates. On review of country-specific data, the prevalence of Asian and African studies generally reported lower prevalence rates than other regions (Europe, North and South America). These studies were largely based upon self-reported data which may suggest under-reporting of ADHD diagnosis which result in under recognition. Cultural or institutional factors related to concerns of disclosing the diagnosis including mental health stigma may contribute and further research may be helpful to understand this.

### Methods of assessment

The wide range of prevalence reported makes it difficult to estimate the prevalence of ADHD. While validated tools like the ASRS may improve diagnostic reliability there is much variance between studies and assessment methods. In the study by Mattos et al. in Brazil, it was shown that significant difference in diagnosis result from the use of ASRS compared with the more comprehensive KSADS assessment and the KSADS with probing with decreasing rates of diagnosis. This also suggests that screening methods may over-estimate the presence of ADHD, and that a more structured assessment may yield a more accurate diagnosis. Interestingly, there is significant variability in prevalence between studies done within the same country. For example, in Brazil, Mattos et al. reported 37% screened positive on the ASRS while Fasanella reported 3.8% prevalence with self-reporting. In Saudi Arabia, the reported prevalence ranged from 5.78% with self-reporting, to 33.5% with ASRS > 17 and WURS > 46, to 38.9% with the ASRS screener. This discrepancy does highlight the difficulties in diagnosis and screening of ADHD, with the possibility of high degrees of false positives when the ASRS is used. Only Rapinesi et al. used the Structured Clinical Interview for DSM-5 (SCID) and reported a prevalence of 16.3% of medical students in Italy. There were no trends with regards to countries or continents which we were able to draw from this study, and prevalence does appear to range higher than the prevalence listed in the general population. Some weight also needs to be given to how cultural factors, stigma, and disclosure norms also influence reporting and diagnosis. In some contexts, there may be concerns of perceived professional repercussions discouraging disclosure, while in others, support frameworks encourage openness. Cross-national differences in medical education policies, disability law, and the availability of specialist assessments must therefore be considered when interpreting prevalence data.

Future research should prioritize objective and structured assessments such as the DIVA or the Connors Adult ADHD Diagnostic Interview alongside self-reporting.

### Need for early screening and intervention

Currently, medical schools largely utilize self-report and there is limited support for students with ADHD. Compounding this issue, there are few structured means of screening and supporting medical students with ADHD. Students with undiagnosed ADHD may suffer the symptoms without ever seeking formal diagnosis or realizing that their impairments may be treatable. Students with ADHD reported experiencing bullying and isolation, and when diagnosis was disclosed, had no means of personalized support in medical school (22). This being said, it is important to position this within the focus on wider disability accommodations for medical students. Conditions such as dyslexia, depression, anxiety, and other learning or psychiatric disorders also impact student success. Our findings should therefore be interpreted within a broader conversation on inclusive medical education, recognizing that comprehensive student support must extend beyond single-condition screening. A tiered approach—initial broad screening for wellbeing and learning needs, followed by targeted assessments for conditions such as ADHD—may be more equitable and resource-efficient.

Our review adds to this discourse by illustrating how differences in diagnostic practices and disclosure environments shape reported prevalence. Future work should integrate prevalence data with qualitative insights into students’ lived experiences and barriers to care, as these factors are critical to developing responsive support systems.

Given the cognitive and emotional demands of medical training, early screening for ADHD is important for medical education. Identifying students with ADHD allows for early treatment and tailored support. Given that ADHD is easily treatable through pharmacological and non-pharmacological means, this potentially will improve academic and professional performance and reduce burnout risk. Screening positive for ADHD may be part of a tiered screening for treatable mental disorders. Institutions should consider incorporating ADHD screening, psychiatric and psychological support and providing accommodations for affected students to improve mental wellness in the cohort, as well as improve student performance. This is especially so given that up to one third of students report symptomatic attention deficit symptoms.

### Strengths and limitations

This review synthesizes findings from diverse populations and methodologies, offering a comprehensive overview of ADHD prevalence among medical students. However, limitations include heterogeneity in study designs and potential biases in self-reported data. There were few studies with structured objective assessments of ADHD, and most relied upon the brief ASRS screener which is not diagnostic but rather a screener or suggest ADHD symptoms. Additionally, the exclusion of non-English studies may limit generalizability. Publication bias may also contribute to studies with higher prevalence being published, resulting in the studies reporting higher prevalence being published. Future work may include meta-analytic assessments of the data and cost-effectiveness and academic outcomes of screening in the medical student population.

## Conclusion

This systematic review highlights the varying prevalence of ADHD among medical students, the challenges in assessment methods, and the critical need for early intervention. While ADHD is an important contributor to student distress and functional impairment, it should be addressed within a comprehensive framework for identifying and supporting a range of cognitive, learning, and mental health conditions in medical education. Recommendations for medical schools include adopting tiered screening processes, integrating structured diagnostic assessments, and strengthening institutional capacity to provide tailored, stigma-free support. Addressing the undiagnosed and symptomatic ADHD population can improve student well-being and academic outcomes, and may be an important mitigating factor in addressing burn out, medical errors and suicide risk in medical students and future trainees. Future research should combine quantitative prevalence estimates with qualitative analyses to inform culturally sensitive, policy-aligned approaches to disability support in medical training.

## Data Availability

The original contributions presented in the study are included in the article/supplementary material. Further inquiries can be directed to the corresponding author.
